# Risk-taking neonates do not pay a survival cost in a free-ranging large mammal, the fallow deer (*Dama dama*)

**DOI:** 10.1098/rsos.220578

**Published:** 2022-09-21

**Authors:** Bawan Amin, Laura Verbeek, Amy Haigh, Laura L. Griffin, Simone Ciuti

**Affiliations:** ^1^ Laboratory of Wildlife Ecology and Behaviour, School of Biology and Environmental Science, University College Dublin, Dublin, Ireland; ^2^ Mathematical Institute, Leiden University, Leiden, The Netherlands

**Keywords:** animal personality, state, pace-of-life, resource allocation, resource acquisition, fallow deer

## Abstract

Recent debate has focused on whether variation in personality primarily reflects variation in resource allocation or resource acquisition of individuals. These two mechanisms predict different relationships between personality and survival. If personality mainly reflects variation in resource allocation, then bold (i.e. risk-taking) individuals are expected to live shorter lives, whereas the opposite pattern is expected with resource acquisition. Here we studied the relationship between neonate personality and early-life survival in 269 juveniles of a population of fallow deer (*Dama dama*). We found that bolder individuals paid no apparent survival cost. Interestingly, among-individual differences in the physiological response at capture (heart rates, which covary with the behavioural response, i.e. latency to leave) were linked to survival, where individuals with lower heart rates when handled by humans had a higher probability of early-life survival. This suggests that bolder individuals may be of higher state than their shyer counterparts. As the first study linking neonate personality to survival in a free-ranging mammal, we provide novel insights into drivers behind early-life individual variation.

## 1. Introduction

**Figure 1.**
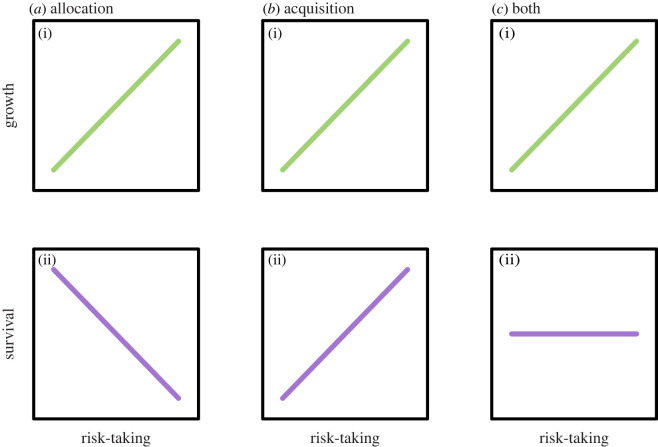
Alternative scenarios of the relationship between risk-taking behaviour and resource allocation versus acquisition and the patterns they generate between behaviour and growth (i), and between behaviour and survival (ii). Under every scenario, risk-taking behaviour is associated with increased growth (i). However, each scenario generates a different pattern between behaviour and survival. In (*a*), among-individual differences in behaviour relate mostly to differences in resource allocation. Individuals that display more risk-taking behaviour allocate more towards current reproduction and therefore have increased growth, but reduced survival. In (*b*), among-individual differences in behaviour relate mostly to differences in resource acquisition. Individuals that display more risk-taking behaviour acquire more resources and are therefore able to maintain high state, leading to higher growth and survival. In (*c*) among-individual differences in behaviour relate to differences in resource allocation and acquisition. In this case, risk-taking individuals will also have increased growth, but without the cost of survival. A trade-off between behavioural types, where both extremes (bold vs shy) have equal reproductive output overall is only present under scenario (*a*), whereas under scenarios (*b*) and (*c*) risk-taking individuals acquire or maintain higher overall quality without paying any survival costs. Figure is largely inspired by Laskowski et al. [4].

Individuals within a population tend to differ consistently among each other in their average behaviour, and these among-individual differences (i.e. animal personality) have been shown to play a major role in ecology and evolution [1]. How these differences arise and are maintained in evolution, however, remains an unsolved question. One of the most prominent hypotheses, the *extended* pace-of-life syndrome (POLS) hypothesis (see [2]), theorizes that life-history trade-offs maintain the variation in personality. Within this framework, individuals are expected to covary in their behavioural, physiological and life-history traits [2,3]. The trade-off in resources allocated to current versus future reproduction has been suggested as an underlying driver of these covariations. Under this model, individuals exhibit their own pace-of-life (POL), along the slow-fast continuum, depending on how much of their resources they allocate to either current or future reproduction [3]. Within the extended POLS, individuals with a fast POL are expected to show risk-taking behaviour, which in turn leads to faster growth, at the cost of mortality ([2]; figure 1*a*). These fast POL individuals are thought to allocate most of their resources towards current reproduction, whereas individuals with a slow POL are expected to show the opposite patterns by allocating most of their resources to future reproduction, i.e. risk-averse behaviour, slow growth rates, but higher survival [2,3].

The empirical support for patterns predicted under the extended POLS, however, have so far been ambivalent at best [5–7]. Especially when it comes to survival, there has been no support for the POLS prediction that bolder (i.e. more risk-taking) animals have shorter lifespans in recent meta-analyses [6,7]. Haave-Audet *et al.* [7] found no relationship between personality and survival, whereas Moiron *et al*. [6] reported weak evidence that bolder animals actually survive longer, but this was only the case in wild populations, highlighting the importance of ecology for these patterns. This suggests that bold individuals may be of higher quality in general [6], due to their ability to systematically gain more resources than shy individuals [4]. This would enable them to consistently allocate more resources to both current and future reproduction, since they would have more resources overall, which could lead to higher reproduction *and* survival ([4]; figure 1*b*). There is indeed accumulating evidence that the relationship between personality and survival is mediated by variation in resource acquisition, and not so much by resource allocation [4,6,7].

This relationship between personality and resource acquisition is not new and has been previously suggested as a driver of both short- and long-term behavioural consistency under state-dependent models [8]. Under this framework, individuals are expected to express the optimal behaviour based on their underlying state (e.g. body condition, size and energy reserves). Behavioural consistency in the long term is then maintained by positive feedback loops between state and behaviour [8,9], and thus, these models theorize that variation in animal personality is a consequence of variation in the initial state [8]. Under neutral ecological conditions, individuals that are in a high state are expected to behave boldly, and this risk-taking behaviour then maintains their high state due to higher resource acquisition [8]. Low-state individuals, on the other hand, will not be able to behave boldly and therefore make the ‘best out of a bad situation’ by being shy, i.e. risk-averse [8].

Models revolving around among-individual differences in resource allocation (such as POLS), and models revolving around among-individual differences in resource acquisition (such as state-dependent personality models), are not mutually exclusive and even overlap. Both frameworks, for instance, predict a positive relationship between boldness and growth—a prediction which is supported by empirical data [5,10]. Where the two types of models differ, however, is in the relationship between personality and survival [4]. If resource allocation is the driver of among-individual patterns, then bold individuals will have shorter lifespans, either due to higher predation or injury risk as a consequence of their risk-taking behaviour, or due to the higher metabolic costs of faster growth rates [2,3]. If, however, resource acquisition is the primary driver of the relationship between personality and survival, then bolder animals are expected to have a similar or even longer lifespan than shy individuals due to their increased amount of resources acquired [4,7]. Therefore, we can provide evidence for either resource allocation or resource acquisition as the main driver of variation in personality, by studying the relationship between behaviour and survival.

Although an increasing number of studies have already investigated this relationship, the majority focus on (sub)adults, with a lack of data on juveniles, especially in free-living mammals. For example, in a recent extensive meta-analysis including 82 studies examining the relationship between behaviour and survival, only three included juvenile free-living mammals either partly or as the main subject of the study [7]. None of these, however, have focused on neonates that were within their first weeks of life, even though studying individuals at their earliest stages of life is crucial to gain a proximate understanding of behavioural variation. Behavioural phenotypes are influenced by the environment and experiences that individuals face over their ontogeny [11]. Neonates have yet to experience much of these, which makes them ideal for studying initial state and behaviour. Furthermore, different selection pressures can apply to different life-stages (e.g. [11,12]), meaning that patterns in adults may not necessarily be applicable to neonates.

Here we aimed to provide insights into the drivers of personality in juveniles of a free-ranging mammal, of which there is a paucity of data, by investigating the relationship between initial state (i.e. birthweight), neonate personality (i.e. neonate response to capture) and early-life survival of fallow deer (*Dama dama*) fawns. Fallow deer are an excellent fit-for-purpose study species, since neonates display repeatable among-individual differences within days of being born [13]. Amin *et al*. [13] have shown how neonates of this population vary in their coping with human captures in both their physiological (i.e. heart rates at the end of capture) and behavioural (i.e. latency to leave upon release) response. Furthermore, these responses are phenotypically correlated, with bold (i.e. risk-taking) individuals having lower heart rates and higher latencies than shyer individuals [13]. There is also evidence that neonate personality is related to resource acquisition months later, with individuals that displayed bold behaviour at capture spending less time scanning, and thus having more time to acquire resources [14].

Here we specifically tested the relationship between (i) initial state (taking birthweights as proxy) and early-life survival and (ii) neonate personality and early-life survival. Even though the physiological and behavioural neonate responses have been shown to covary at the unpartitioned phenotypic level [13], we decided to include both metrics in our analysis since a concurrent study suggests that they can be measuring different traits [14]. For our first objective, we predicted that neonates with a higher birthweight would be in better condition and, therefore, that they would have a higher chance of survival. For our second objective, we had two separate hypotheses. If among-individual variation in *resource allocation* is the main driver of personality (as predicted under the extended POLS), then we would expect bold individuals to have lower survival than shyer individuals ([4,7]; figure 1*a*). If, however, patterns between behaviour and survival are mainly driven by among-individual differences in *resource acquisition*, as state-dependent models predict, then we would expect that bold individuals will not pay any survival costs and survive even better than shy individuals ([4,7]; figure 1*b*).

## 2. Methods

### 2.1. Study site and population

We conducted this study in Phoenix Park, a 7.07 km^2^ urban park (approx. 80%: open grassland, approximately 20% mixed woodland) located in Dublin, Ireland. Within this park, there is a resident population of free-ranging fallow deer, consisting of approximately 600 individuals in autumn, after the birth of fawns. There is a natural segregation between adult sexes, causing adult bucks and adult females to spend most of the year spatially separated [15]. Neonates are captured within their first weeks of life (mean age ± s.d.: 3.6 ± 2.4 days at first capture and 7.9 ± 3.8 days at recaptures) and ear-tagged with colour coded tags annually in June, when most births occur. As a result of this marking procedure, an estimated 80% of the population is individually recognizable. Fallow deer are a hider species and fawns remain hidden, usually in tall grass or understory vegetation, away from the main doe herd during the first two–three weeks of life after which they are brought into the doe herd by their mothers [16,17]. Fawns are occasionally predated upon by red foxes (*Vulpes vulpes*), the only natural predator in the park, and domestic dogs who are brought into the park by visitors. Deer are culled annually by professional stalkers over the winter period as part of the population management led by the Office of Public Works.

### 2.2. Neonate personality at capture

As a part of monitoring and management of the deer in the park, neonate fawns have been routinely captured and ear-tagged with plastic tags (Allflex medium, Mullinahone Co-op, Ireland) on an annual basis in June since the 1970s [18]. Fawns were located by patrolling geographical areas typically used by does as fawning sites. After location, fawns were captured and immobilized using fishing nets (1–1.5 m diameter; various brands). We tagged a total of 285 fawns over 3 consecutive years (*n* = 102 in 2018, *n* = 83 in 2019 and *n* = 100 in 2020), of which 137 individuals were recaptured at least once. We recorded the following covariates which have been shown to affect neonatal response to handling [13]: weight (in kg) was measured using a digital scale by laying the fawn in a 100 l bag (resolution: 0.01 kg – Dario Markenartikelvertrieb, Hamburg, Germany); air temperature was measured at the bed-site location using a digital thermometer (Grandbeing, China). Prior behaviour of the fawn, i.e. its alertness, was scored on a scale from 0 (inactive) to 1 (active), following Amin *et al*. [13].

As measures of neonate personality, we selected a physiological trait (heart rates prior to release, i.e. the physiological response of fawns to human handling) and a behavioural trait (latency to leave upon release). These were both shown to be repeatable at the individual level, with yearly repeatability estimates ranging from 0.25 to 0.39 [13]. Heart rates were taken directly before the weighting of the fawns (which is the last measurement prior to release) and quantified by counting the number of beats per 20 s using a Lightweight Dual Head Stethoscope (MDF®, California, USA). The latency to leave (in seconds) on release was defined as the time it took the fawn to stand up after opening the weighing bag. We took 10 s as the maximum value and assigned that to individuals that had not moved before then [13]. For further details of the neonate captures and the capture protocol, see Amin *et al*. [13].

### 2.3. Survival data

We acquired survival data by analysing every individual sighting from data collected between June 2018 and the start of May 2021. The fallow deer population in Phoenix Park was monitored by members of the UCD Laboratory of Wildlife Ecology and Behaviour on a weekly basis by surveying all sectors of the park systematically, following the protocol defined by Griffin *et al*. [19] in addition to concurrent core data collections aimed at monitoring the entire population during key periods of the annual biological cycle (e.g. weaning, rut, antler growth and shed). In total, we collected 19 048 observations of the 285 fawns over the 3 years of study. Due to the large extent of our sample size, we inferred mortality when an individual was not sighted for at least six months without being seen at least twice afterwards to account for human error sightings. In those cases, an individual's last sighting was taken as their potential date of death. Since certain individuals had missing values for some of the variables we used in our models (see statistical analysis), we had to omit them from our analysis. Therefore, the final sample size consisted of 269 individuals, of which 176 were alive and 93 dead (table 1). Out of these 93 dead individuals, 30 were inferred to be dead since they were not resighted after a certain date, whereas 63 individuals were found dead over the course of this study. In the cases where an individual was found dead, we used the date on which their carcass was found as the death date.

**Table 1.** Overview of the individually recognizable fallow deer fawns that were monitored for survival over the period of June 2018–May 2021. ‘Still alive’ and ‘dead’ refer to the survival status of the fawns by May 2021, ergo a different monitoring period for fawns belonging to different cohorts.

cohort	individuals	still alive	dead	time observed
2018	93	45	48	31 months
2019	80	50	30	21 months
2020	96	81	15	9 months
total	269	176	93	—

### 2.4. Statistical analysis

To study the relationship between survival, initial state (birthweight) and neonate personality, we ran a cox proportional hazards model. We report below a step by step explanation on how we estimated birthweights for each individual (section ‘predicting birthweights’), how we computed neonate response at the among-individual level (section ‘neonate personality’) and how we built and ran the survival model (section ‘survival model’). Finally, we wanted to study the relationships between our neonate traits (i.e. birthweight, heart rate and latency to leave; section ‘covariation between neonate traits’). Although we have shown previously that heart rate and latency to leave covary [13], this relationship was shown at the phenotypic level [13], whereas here we wanted to study such a relationship at the among-individual level. All analyses were performed in RStudio (Version 1.3.1093) using R version 4.0.2 (R Core Team, 2021).

### 2.5. Predicting birthweights

In order to calibrate the capture weights of the fawns to a comparable birthweight (i.e. day 1 estimate), we ran a linear mixed-effects model (lme4-package; [20]), which was then subsequently used to predict birthweights for each individual fawn, following the same protocol as Griffin *et al*. [19]. We used the weights as the response variable, the estimated age in days at capture (both linear and quadratic term) as a fixed factor, and included fawn ID as a random intercept. We ran this model on data collected over 3 subsequent years, which consisted of a total of 459 captures on 275 individuals. Our model explained most of the variation in weight ($R_{\mathrm{marginal}}^{2}=0.56$; $R_{\mathrm{conditional}}^{2}=0.93$), with weight at capture being mostly explained by our estimate of age and individual variation (i.e. the random intercept). We therefore used this model to predict the birthweight of the fawns that we have included in our survival analysis.

### 2.6. Neonate personality

Because of the inability to incorporate repeated measures into covariates of a Cox proportional hazards model, we opted to use the best linear unbiased predictors (BLUPs) extracted from univariate models for both the heart rates as well as the latency to leave at capture. For both these univariate models, we used the same model structure as in our previous study (see [13] for more details on how these model structures were determined). For the heart rates, the model included prior behaviour (both linear and quadratic term), time of the day (in hours), weight and air temperature as explanatory variables. For latency to leave, the model included the capture number, prior behaviour and weight (both linear and quadratic term) as explanatory variables. Fawn ID was used as a random intercept for both models. To improve model convergence, all numerical explanatory variables were scaled prior to analysis, such that each variable was centred at their mean value and standardized to units of 1 phenotypic standard deviation. Full details of the univariate models are given in the electronic supplementary material S1.

The use of BLUPs in behavioural ecology has been criticized before, due to uncertainty around the mean not being taken into account [21,22]. As a solution, it has been proposed to take forward the uncertainty, by using *a posterior* distribution instead of just the BLUP [21]. A recent study using simulations has indeed shown that such a solution reduces uncertainty, but in the process introduces a negative bias [23]. We therefore have decided to use the BLUPs as a measure of among-individual responses, which have increased uncertainty but do not suffer from this systematic bias. To further support the robustness of our results, we also ran a supplementary analysis using the raw phenotypic values of the first captures only, instead of the BLUPs (electronic supplementary material S3). This additional analysis yielded similar results to our main analysis, indicating that our patterns are robust and not generated by the use of BLUPs.

### 2.7. Survival model

We modelled mortality risk by running a Cox proportional hazards model using the *survival* package ([24]; see electronic supplementary material S2 for the R-markdown file of the analysis). The model included predicted birthweight (full details above), heart rate and latency to leave at capture (both as BLUPs, see above) as main predictors. In addition, we included birthyear to correct for among-year differences (categorical, 3 levels), sex (categorical, 2 levels) and birthdate (categorical, 3 levels, i.e. early, mid and late fawning season; separated based on equal number of observations) as fixed factors due to their potential as confounding variables. Individuals that were still alive by the end of the data collection were right-censored [25], but this was at a different moment in the lifetime for each cohort due to the nature of our study (table 1). The proportional hazards assumption was checked and the Schoenfeld residuals were plotted. Additionally, the linearity of the variables and the presence of outliers in the data were checked using the *survminer* package [26]. In all cases, model assumptions were successfully met. Survival plots were also made using the *survminer* package [26], which were used for visualization purposes only. We considered an effect statistically clear when the 95% confidence interval of the adjusted hazard ratio (AHR) did not overlap with 1.

### 2.8. Covariation between neonate traits

As mentioned above (section ‘neonate personality’), we used the BLUPs as estimates of the heart rate and latency to leave at the among-individual level. To investigate the relationship between these two traits at the among-individual level, however, we were able to run a bivariate MCMC-model since we had repeated measures of both responses. This method has been validated before and is suggested to be more appropriate for estimates at the among-individual level due to partitioning total variance into within- and among-individual variance [21,22]. As explanatory variables, we included all the variables that were included in the separate univariate models, used to estimate the BLUPs (see section ‘neonate personality’ above; electronic supplementary material S1, S4). We used a weakly informative prior, [R = list(V = diag(2), nu = 0.002; G = list(G1 = list(V = diag(2), nu = 1.002)))], following our previous study [14]. We ran our MCMC-chain for a total length of 1 050 000 iterations, with a thinning of 500 and a burnin of the first 50 000 iterations, leading to a total of 2000 saved iterations. Model convergence was checked by running three additional chains and calculating the multivariate scale reduction factor [27], which we estimated at 1.1. We also visually inspected the chains, ensuring that every parameter had an effective sample size of at least 1500, and the autocorrelation of the posterior means and variances. From these, we concluded that the chains had converged properly and had negligible autocorrelations. We also ran a supplementary analysis where we estimated among-individual covariance between these two traits using the BLUPs, with very similar results (electronic supplementary material S4).

Finally, we examined the relationship between our estimate of birthweight and our neonate personality measures by estimating the Pearson correlation coefficient between birthweight and the BLUPs of the neonate responses. The full analysis, including code, of the bivariate MCMC-model and the correlations between birthweight and the neonate personality BLUPs is given as electronic supplementary material, S4.

## 3. Results

**Figure 2.**
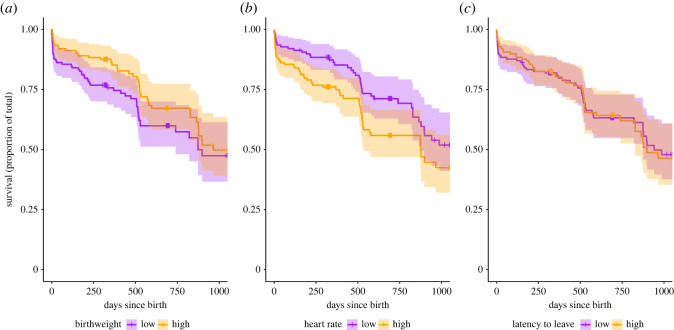
Survival plots visualizing the effects of (*a*) birthweight, (*b*) heart rates at capture and (*c*) latency to leave at capture on the early-life survival of fallow deer fawns. Lines are given with 95% confidence intervals to indicate uncertainty. Marks on the lines indicate right-censored individuals, with clusters around day 320 and 690 indicating the end of observation time for cohort 2020 and 2019, respectively (table 1). Statistical inferences cannot be made based on this figure. Categorical distinction into high and low, with the median value as divider, has been made for visualization purposes only. All inferences have been made on the model output (table 2), which modelled the effect of these three variables on early-life survival on a continuous scale.

We found that birthweight had a statistically clear positive effect on survival (table 2; figure 2*a*). Individuals that were heavier at birth had a lower risk of early-life mortality (AHR = 0.73, 95% CI [0.55, 0.97], *p* = 0.032, *n* = 269). In terms of neonate personality traits, we found that higher heart rates were positively associated with mortality (table 2; figure 2*b*). Individuals with higher heart rates at capture had an increased risk of early-life mortality (AHR = 1.40, 95% CI [1.00, 1.94], *p* = 0.048, *n* = 269). There was, however, no statistically clear effect of the behavioural response, i.e. latency to leave, with early-life survival (table 2; figure 2*c*). We also found no statistically clear effect of our other explanatory variables, i.e. birthyear, sex and birthdate, on early-life mortality risk (table 2).

**Table 2.** Summary table of the Cox proportional hazard model. We have given the AHR ± 95% CI for each explanatory variable in our model. AHR values below 1 indicate a decreased risk of mortality, whereas AHR values above 1 indicate an increased risk of mortality for each increasing unit of the responding explanatory variable. Additionally, we have also provided the *z*-value for each variable. The model was run on a sample size of 269 individuals of which 176 were still alive by the end of the sampling period. Statistically clear effects are displayed in italics.

explanatory variables	AHR	[95% CI]	*z*-value
*birthweight*	*0.73*	*[0.55, 0.97]*	*−2.14*
*heart rate*	*1.40*	*[1.00, 1.94]*	*1.98*
latency to leave	1.21	[0.66, 2.22]	0.62
birthyear (2019)	0.86	[0.52, 1.44]	−0.56
birthyear (2020)	0.74	[0.38, 1.44]	−0.89
sex (m)	1.16	[0.76, 1.78]	0.68
birthdate (mid)	1.24	[0.75, 2.07]	0.83
birthdate (late)	1.49	[0.89, 2.50]	1.50

There was a clear negative covariation between heart rate and latency to leave at the among-individual level, estimated from our bivariate MCMC-model (*r* = −0.38, 95% HPD [−0.57, −0.18], *n* = 276). The supplementary analysis, using the BLUPs, supported this and led to a similar estimate (electronic supplementary material S4). We further found a weak, non-significant trend between birthweights and heart rate (*r* = −0.11, 95% CI [−0.22, 0.01], *p* = 0.08, *n* = 269), whereas there was no clear relationship between the birthweight and the latency to leave (*r* = 0.09, 95% CI [−0.03, 0.21], *p* = 0.14, *n* = 269).

## 4. Discussion

Although animal personality is a widespread phenomenon that plays a key role in ecology and evolution [1], the underlying drivers of this variation are still subject to debate. This is mainly centred on whether resource allocation or acquisition is the main driver of among-individual differences in behaviour [4]. Differentiation between these two can be made by relating personality to survival ([4,7]; figure 1). Models assuming differences in resource allocation to be underlying behavioural differences expect trade-offs between behavioural types [2], whereas models assuming resource acquisition as underlying driver expect bold individuals to be better off, due to differences in initial state [8]. Here we present empirical data on this matter, in a population of free-ranging fallow deer. We found that individuals with (i) a higher birthweight—our proxy for initial state—and (ii) lower heart rates at capture had a higher probability of early-life survival, whereas there was no clear effect of the behavioural response of neonates on survival. Altogether, our results provide no evidence for among-individual differences in resource allocation as the main driver of variation in early-life personality, but rather suggest that bold individuals may be of better state.

Birthweights have been shown to be positively associated with early survival in many different species in a broad range of taxa (e.g. [28–30]), making it a suitable proxy for initial state. Our results support these previous findings; with every kg increase in birthweights, fawns had a 27% increase in survival. Although birthweights can be related to abiotic factors [31], there is a robust body of evidence that connects offspring birthweight to maternal condition or traits [19,32–34]. In this population specifically, Griffin *et al*. [19] have previously shown how among-individual differences in maternal behaviour are associated with fawn birthweights. Mothers that have increased interactions with park visitors, i.e. beg for food more, tend to deliver fawns with higher birthweights [19]. Griffin *et al*. [19] suggested that this effect on fawn birthweight could imply artificial selection through human wildlife interactions, if birthweights are associated with survival, an effect that we provide support for here.

During stressful situations, individuals tend to have an increase in the activity of the hypothalamic–pituitary–adrenal (HPA) axis. The HPA-axis causes an increase in circulating glucocorticoid (GC) levels, which in turn also leads to higher cardiac activity [35]. Individuals typically differ in the strength of this response, which is usually related to their behavioural response [36]. Here we investigated whether among-individual differences in heart rates during capture were related to early-life survival. High heart rates were previously shown to be associated with high chronic hair cortisol levels and a shy behavioural response, i.e. low latency to leave [13]. Individuals with a strong physiological response are therefore more stress-sensitive, since they adopt a more active coping response during capture and handling by humans, a potential predator. We show that neonates with high heart rates during capture had a lower probability of survival. This indicates that individuals that are more stress-sensitive, i.e. have a stronger physiological response during capture, are less likely to survive. Previous studies have shown a similar pattern in juveniles of other species, where high levels of GC were also negatively associated with survival. In white-tailed deer (*Odocoileus virginianus*) for instance, higher baseline salivary cortisol was associated with lower survival at 12 weeks, although sample sizes were very low [37]. Similarly, juvenile European white storks (*Ciconia ciconia*) that had higher levels of blood GCs also had lower survival rates [38]. Here we have shown that these effects are also present at the among-individual level in neonates.

Boldness has been suggested to be associated with increased mortality [39–42]; by being bold, individuals are more exposed to risks of injury or predation. Recent meta-analyses have, however, failed to confirm that boldness is associated with lower levels of survival [6,7], and there is even ample evidence that it can be associated with higher levels of survival [43–46]. Still, very little is known about how these patterns apply to neonates of free-living mammals, a study group that has rarely been focused upon. In the context of this study, we found no clear effect of among-individual differences in the behavioural response of neonates on survival. Individuals that behaved boldly did not suffer from any survival costs. This is contrary to what is predicted under models where resource allocation is the main driver of behavioural differences [4]. It is possible that the lack of a clear negative pattern between survival and bold behaviour in our study is due to the low predation risk in this population, since predation has been shown to be an important factor in relationships between behaviour and survival [43,47]. It is, however, important to note that under the resource allocation models, boldness should still be expected to be negatively related to survival even with low predation, since bold individuals are supposed to allocate fewer resources to survival in general [2]. Therefore, we found no support for resource allocation as the main driver of among-individual variation in neonate fallow deer.

**Figure 3.**
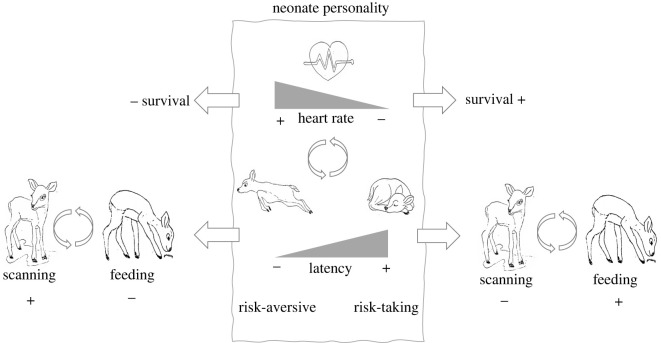
An overview of the complex relationships between neonate personality, resource acquisition and survival. Neonate physiological (i.e. heart rates) and behavioural (i.e. latency to leave) responses to human capture and handling are repeatable and negatively correlated to each other (central panel). Despite their correlation, both are related to different aspects of later behaviour or life history. The behavioural response is related to behaviour later in life, where bold neonates spend less time scanning and have thus more time for resource acquisition (lower right; [14]), and shy neonates spend more time scanning and have less time for resource acquisition (lower left; [14]). The physiological response is related to survival, where higher heart rates are negatively associated with survival (upper half; this study). Altogether, this overview shows how personality can affect resource acquisition, with underlying physiological patterns suggesting that personality is also related to state.

The physiological (i.e. heart rates) and behavioural (i.e. latency to leave) response of neonate fallow deer at capture covary, both phenotypically [13] as well as at the among-individual level. It is therefore surprising that we found here that there was only a clear relationship between survival and the physiological, but not the behavioural, response. Furthermore, the behavioural response of these neonates has been previously shown to be related to among-individual differences in time spent scanning months later, allowing bolder individuals to spend more time on resource acquisition [14]. The lack of a pattern in the behavioural response with survival suggests that the survival patterns reported here are not due to differences in predator exposure or predator interactions. Rather, since survival was related to the physiological response, our findings imply that this response may be linked to the internal state of individuals. Since lower heart rates were related to both higher survival and bolder behaviour, this suggests that boldness may be positively associated with state, as predicted by state-dependent theory [8]. The findings presented here, in combination with previous work on neonates in this population [13,14], provide a mechanistic overview of how personality can affect life history (figure 3).

In conclusion, we have studied the relationship between neonate personality and survival for the first time in a free-ranging mammal population, aiming to provide novel insights into the drivers of variation at the individual level. We found no clear direct relationship between bold behaviour and survival, indicating that boldness did not come with a survival cost. We therefore provide no support for resource allocation as the main driver behind among-individual differences. Interestingly, we did find that lower heart rates were related to both higher survival and bolder behaviour. These patterns suggest that, in this context, bold neonates may have higher state, and therefore be of higher quality than their shy conspecifics. Future studies should aim to investigate how general these patterns are, and how they may be affected by different ecological conditions and selection pressures.

## Data Availability

We fully support open and transparent science. Therefore, the data file and R-markdown files of the analysis are uploaded as electronic supplementary material [50].
